# Bio-stimulating effect of endophytic *Aspergillus flavus* AUMC 16068 and its respective ex-polysaccharides in lead stress tolerance of *Triticum aestivum* plant

**DOI:** 10.1038/s41598-024-61936-0

**Published:** 2024-05-25

**Authors:** Hend A. EL-khawaga, Abeer E. Mustafa, Maie A. El khawaga, Amira Y. Mahfouz, Ghadir E. Daigham

**Affiliations:** Botany and Microbiology Department, Faculty of Science, Al-Azhar University, (Girls Branch), Cairo, Egypt

**Keywords:** *Aspergillus flavus*, Exopolysaccharide, Endophyte, Lead toxicity, Wheat plant, Microbiology, Plant sciences

## Abstract

Heavy metal accumulation is one of the major agronomic challenges that has seriously threatened food safety. As a result, metal-induced phytotoxicity concerns require quick and urgent action to retain and maintain the physiological activities of microorganisms, the nitrogen pool of soils, and the continuous yields of wheat in a constantly worsening environment. The current study was conducted to evaluate the plant growth-promoting endophytic *Aspergillus flavus* AUMC 16,068 and its EPS for improvement of plant growth, phytoremediation capacity, and physiological consequences on wheat plants (*Triticum aestivum*) under lead stress. After 60 days of planting, the heading stage of wheat plants, data on growth metrics, physiological properties, minerals content, and lead content in wheat root, shoot, and grains were recorded. Results evoked that lead pollution reduced wheat plants’ physiological traits as well as growth at all lead stress concentrations; however, inoculation with lead tolerant endophytic *A. flavus* AUMC 16,068 and its respective EPS alleviated the detrimental impact of lead on the plants and promoted the growth and physiological characteristics of wheat in lead-contaminated conditions and also lowering oxidative stress through decreasing (CAT, POD, and MDA**)**, in contrast to plants growing in the un-inoculated lead polluted dealings. In conclusion, endophytic *A. flavus* AUMC 16,068 spores and its EPS are regarded as eco-friendly, safe, and powerful inducers of wheat plants versus contamination with heavy metals, with a view of protecting plant, soil, and human health.

## Introduction

Soil is a natural environment for diverse microbial species that can become polluted by heavy metal accumulation. As a result of soil pollution, which has become a severe problem in many nations, agricultural land around the world is declining^1^. The application of low-quality waters (wastewater) for irrigation in agriculture, unrestricted and untreated xenobiotic pollution discharge, and fast-expanding industries all pose severe, intolerable risks to the viability of agroecological regions^2^. After accumulating in the soil, heavy metals invade the food chain and are then transmitted to the final consumers, where they cause health issues in people^2,3^. However, the toxic effects of heavy metals that enter vegetal tissues can impair some physiological reactions in plants, including wheat^4^, and adversely affect human health^5^. Briefly, extremely high concentrations adversely affect plants by modifying membrane permeability^6^**,** blocking physiologically active enzymes^7^, deactivating photosystems^8^ and disturbing mineral metabolism^9^**.**

Common wheat, (*Triticum aestivum* L.), is an important strategic cereal grain that, particularly in developing countries, is essential to the traditional human health system. For about 36% of the world’s population, wheat is the primary staple food crop. Wheat is susceptible to heavy metal accumulation, like many other plants^10^. Once heavy metals are taken by plants, the stress causes a variety of reactions in them, including germination, growth, metabolic reactions, and yield losses in wheat^5,11^.

In consequence, the accumulation of ROS in plant tissues results in electrolyte leakage and compromises the integrity of the cell wall^12^. Cell death is typically the result of the disruption of the oxidation of membrane proteins and lipids^13^.

A plant biostimulant is a substance or microbe that plants manage to boost nutritional efficacy, stress tolerance, and/or crop quality traits, regardless of the amount of nutrients present^14^. Also, Biostimulants improve plant adaptability for preserving sustainable yields from agriculture and enhancing soil fertility and structure^15^, and they are seen to be a viable strategy for stress management^16^**.**

Extracellular polymeric compounds have been a topic of interest for a long time because of their ability to decay, biocompatibility, and capabilities for thickening, gelling, and emulsion^17^. Fungi and bacteria support soil structure stability by creating extracellular polymers and decomposing aromatic humic components, which result in clay-metal–organic matter complexes. Fungi support as well, albeit with less persistence, by attaching particles through hyphae^18^**.**

Endophytes are microbial organisms that colonize the inter and intracellular spaces of higher plant tissues without inflicting obvious destruction on the plants in which they live. Endophytes have been demonstrated to be rich in bioactive natural compounds^19^. Endophytes may defend and sustain their host plant by creating a variety of chemicals that, once isolated and defined, may have potential applications in agriculture, medicine, and industry^20^.

Fungal endophytes have attracted widespread interest in recent years due to their previously unknown and distinct features, as well as distinct secondary metabolites with bioactive properties that help them maintain their mutualistic relationship with hosts and increase host tolerance to various biotic and abiotic issues^21^. Polysaccharides are long-chain carbohydrates formed through the complicated polymerization of diverse neutral sugars or uronic acids via glycosidic linkages and are among the most crucial active components in fungi^22^. Exopolysaccharides (EPSs) are a type of carbohydrate released by several bacteria and fungi, including pathogenic and symbiotic bacteria and fungi^23,24^. Exopolysaccharide (EPS) formation is a critical approach for maintaining a humid environment, trapping nutrients, promoting chemical processes, and guarding cells against external conditions, antibiotics, and predatory attacks^17^. As a result, different microbial slimes and EPS are currently studied as aggregate factors in different types of soils for fertility and soil quality. Exopolysaccharide (EPS)- producing microorganisms have developed advanced adaptability and flourishing methods under adverse conditions. The establishment of the cell wall and their capacity to form a large number of solutes are among the improvements they make, which enhances the retention of water and minimizes the impacts of osmotic and ionic stressors. Exopolysaccharide modifies the cell wall construction and may generate a protective biofilm on the root’s surface^25^**.**

Despite some reports here and there, little is known about metal phytotoxicity in wheat and methods to improve the productivity of wheat in metallic-stressed soil. Thus, the goals of this study are to gain insight into the basis of metal toxicity in wheat and how such phytotoxicity might be reduced by introducing microbiological remediation measures into wheat farming practices and explore the efficacy of endophytic *Aspergillus flavus* AUMC 16,068 and its EPS as a prospective biostimulant to reduce the adverse influences of lead metal stress on wheat plants.

## Materials and methods

### Reagents and chemicals

Analytical purity grades reagents and chemicals used in this study were supplied by Sigma-Aldrich Chemical Co (St. Louis Missouri, 63,103, USA). Potato Dextrose Agar (PDA) medium was sourced from Difco (United Kingdom).

### Isolation and purification of endophytes

The endophytic fungal isolates were isolated from the roots and stem of the wheat plant, a wild plant grown in Wadi El-Natroon City, Egypt. The wheat plants were washed with running tap water to eliminate surface dust and then rinsed once more with distilled water. To eliminate epiphytic microorganisms, the plant materials were surface sterilized by immersing in 75% ethanol for 2 min., followed by rinsing with 5% sodium hypochlorite (Na Cl O) for 3 min., followed by washing 3 times with sterile distilled water^26^. Surface sterilized plant sections (0.5–0.3 cm pieces) were placed onto PDA media and incubation was carried out for 7 days at 28 °C under dark conditions. The obtained isolated fungi were purified by the agar streak method and identified morphologically according to their culture features including the texture, color of the colony’s surface, colony growth rate, and reverse pigmentation Then, the microscopic examination of the isolates was applied using (Optika, Italy) light microscope^27,28^.

### Screening the ability of endophytic fungal isolates for Pb (II) maximum tolerance concentration (MTC)

The Pb^2**+**^ tolerance of the endophytic fungal isolates was determined by adding metal ions to the sterilized PDA medium at concentrations from (100, 500, 1000, 1500, 2000, 2500, 3000, 3500, and 4000 ppm). For seven days, plates with three replicates of each concentration and metal-free controls were incubated at 28 °C. By comparing the colony extension radius (mm) of the isolates to the control, the impact of the heavy metals on the isolates’ growth was evaluated and the MTC was calculated. The most palatable fungus isolate was then chosen for additional testing.

### Estimation of exopolysaccharide (EPS) production from the most lead-tolerable endophytic isolates

In this concern, 10% (v v^−1^) of the seed culture of the** f**ungal isolates were inoculated separately in 250 ml flasks containing 50 ml of sterilized medium. The media contains the following ingriedients (g l^−1^): glucose, 40; peptone, 0.5; yeast extract, 1.0; MgSO_4_·7H_2_O, 0.5; KH_2_PO_4_, 0.5. For 14 days, the flasks were incubated at 28 ± 2 °C and 150 rev min^−1^ on a rotary shaker. Next, the EPS was extracted by the method reported by Fang & Zhong^29^**.** Centrifugation at 4 °C (6000× *g*, 15 min.) was carried out to separate mycelia, and the obtained supernatant was mixed with 95% ethanol (in five volumes) and kept at 4 °C for 24 h. The centrifugation of the EPS precipitates was achieved at 8000× *g* for 10 min, then washed three times with distilled water, and dried. Only, the more lead tolerable and EPS producer fungal isolate was selected for further experiments throughout the study.

### Morphological identification of the more lead-tolerable and EPS producer

The most promising EPS producer isolate was identified at the species level using the most documented identification keys and manuals^30–33^. The identification was done by Image Analysis System using Soft-Imaging GmbH software at Applied Mycology Laboratory, Botany and Microbiology Department, Faculty of Science (Boys), Al-Azhar University, Cairo, Egypt. Next, scanning electron microscopy (SEM) of the selected fungus isolate was done using a high vacuum mode of JOEL JSM-5500LV, Scanning Electron Microscope^34–36^**.**

### Molecular identification

The method described by Moubasher^37^ was employed for DNA isolation. The PCR reaction was conducted using Sol Gent EF-Taq in accordance with Al-Bedak and Moubasher indications^38^. The universal primers ITS1 and ITS4 were used for ITS region amplification^39^. For the Aspergillus species under study, a consistent sequence was produced using the DNA STAR software (version 5.05). An outgroup sequence for *Aspergillus wentii* ATCC 1023, one for *Aspergillus sp.* AUMC 16,068 through this investigation, plus 22 sequences from the genus *Aspergillus*: section Flavi acquired from GenBank made up the 24 arrangements in the total ITS dataset used for phylogenetic investigation with the initial settings, MAFFT (version 6.861b) was implemented to align the sequences^40^. Sparse incorrect characters and misalignment gaps were optimized using BMGE^41^. MEGA X (version 10.2.6) was used for maximum-likelihood (ML) and maximum-parsimony (MP) phylogeny studies^42^, and 1000 replications were employed to assess the validity of the reliable conservative trees^43^. The Akaike Information Criterion (AIC) of Model test 3.7 has been used to identify the optimal nucleotide switching model for ML evaluation^44^. The resulting tree was altered and then saved in TIF format following the method of Al-Bedak et al.^45^.

### Characterization of EPS

#### Determination of total sugar content of EPS

The phenol–sulfuric acid method^46^ was used to measure the total sugar content of the most promising EPS producerusing glucose as the standard.

#### FT-IR spectroscopic analysis

The FT-IR spectrum of the EPS was determined in the range of 400–4000 cm^−1^ using an FT-IR spectrometer (Thermo-scientific type, model Nicolet—IS 10, USA)^47^**.**

#### Greenhouse experiments

The pot experiments were meticulously conducted during the winter season of 2021–2022 within a controlled environment situated in a fenced area at the Faculty of Science campus, Al-Azhar University (Girl’s branch), located in Cairo, Egypt. The choice of this location provided an ideal setting for the experiments, ensuring proper supervision and control over environmental factors. To initiate the experiments, soil samples were carefully collected from cultivated farmlands in El-Monofia governorate, known for its diverse agricultural practices. These soil samples underwent a series of preparatory steps to ensure their suitability for the experiment. Initially, the soil samples were air-dried to remove excess moisture and facilitate subsequent processing. Following this, the dried soil was finely ground and sieved through a 2 mm sieve to achieve uniform particle size, in accordance with the method outlined by Piper^48^. The resulting soil material was thoroughly mixed to ensure homogeneity and consistency. The physical and chemical characteristics of the soil samples were then assessed using established techniques^49,50^. This comprehensive analysis included the determination of pH, electrical conductivity (EC), organic matter content, organic carbon content, and lead concentration. The pH of the soil was measured using a pH meter in a soil–water suspension with a ratio of 1:2.5^51^, while EC was determined as described by Kashyap et al.^51^. Organic matter and organic carbon percentages were quantified using a chromic acid titration method following the procedure outlined by Zeng et al.^52^. Lead content in the soil was quantified using Inductively Coupled Plasma (ICP) spectrometry, according to the method outlined by Soltanpour and Schwab^53^. Based on the physical and chemical properties of the soil, as well as the desired concentrations of lead ions, different treatment groups were established to represent varying levels of lead contamination in the soil. This allowed for a comprehensive investigation into the impact of lead stress on plant growth and development. The experimental setup involved the use of porcelain pots, each measuring 37 cm in diameter and 39 cm in height. Individual pots were filled with 10 kg of the prepared soil mixture, providing ample space for plant growth and development. Prior to planting, the soil was enriched with a balanced NPK fertilizer at the recommended proportion of 80 kg N, 30 kg P_3_O_5_, and 15 kg of K_2_O per Feddan (Feddan = 0.4 hectare). These essential nutrients were supplied in the form of ammonium nitrate (33.5%), superphosphate (15.5%), and potassium sulfate (20.5%), respectively. Superphosphate and potassium sulfate were applied before planting, while nitrogen was added at three equal intervals: once before planting and then at three-week intervals thereafter, following the recommended dose of the Ministry of Agriculture, Egypt. This meticulous fertilization regimen ensured optimal nutrient availability throughout the duration of the experiment, promoting healthy plant growth and development.

### *Triticum aestivum* grains

Grains of wheat plants (*Triticum aestivum*) Giza 171 were gained from the Crop Field Institute, Agriculture Research Center, Cairo University. The grains surfaces were sterilized by immersion in 95% ethanol for 5 min., washed 3 times in sterile, deionized H_2_O, followed by soaking in 1.5% solution of sodium hypochlorite for 10 min., and then thoroughly rinsing with distilled H_2_O.

### Inoculation of wheat plants (*Triticum aestivum*) with the more lead-tolerable and EPS-producer fungus

Wheat grains were inoculated with the spores of the EPS producer alone and in combination with their respective EPS. For grains inoculation with fungal spores, 50 gm of the wheat cultivar grains were immersed in 10 ml of selected fungal spore suspension (1 × 10^8^ conidia ml^−1^ distilled water) for 2 h, and the inoculated grains were dried using sterile tissue for 30 min. For grains inoculation with crude (respective) EPS, the fungal spores were cultured in EPS-producing media in an orbital shaker at 150 rpm for 14 days at 28 °C, thereafter centrifuged at 3000 rpm for 15–20 min. This centrifuged filtrate was used for sowing grains. The grains were soaked for two hr. before planting. Control grains were immersed in sterile distilled water. Lead nitrate solution at concentrations of 200, 600, and 1000 mg per kg of soil was added. The control was kept without adding any lead nitrate solution. Before cultivation, the lead nitrate solution had been applied to the soil.

For wheat planting, the treated grains were planted at a depth of 1.5 cm from the soil surface. The pots were irrigated with tap water until the complete germination, and then the plants were thinned to identical 10 plants per pot. The pots were irrigated periodically. During the growth period, both the treated and untreated plants were irrigated with water to maintain the soil at the level of 70% of the field capacity. The field capacity was 1200 ml of tap water. Two weeks following planting, when the seedlings had fully emerged, the seedlings were thinned to a maximum of 15 identical plants per pot.

#### Sampling

Shoot and root samples were obtained to assess and represent the heading stage after 60 days, and the plants were harvested after 130 days from the planting date. Parametric determinations were carried out.

### Growth measurements

#### Shoot lengths

For assessing the morphological parameters, ten plants were randomly selected per treatment. The shoot lengths were measured with the help of a scale (cm/plant).

#### Shoots fresh and dry weights

The plant material was washed several times with tap water, followed by distilled water then weighed accurately and dried in an electric oven at 70 °C (approximately for 24 h), until constant weight was attained, then the dry weights were subsequently calculated as grams per plant.

#### Leaf area

The leaf area (cm^2^/leaf) was determined using the following equation proposed by Quarrie and Jones^54^;

Leaf area = Maximum length × maximum breadth × 76.

### Analytical analyses

For analyses of nutrient concentrations, plant powder was extracted by wet acid digestion in a mixture of concentrated sulfuric acid (H_2_SO_4_), and 30% hydrogen peroxide (H_2_O_2_), as described by Jones^55^. The digested samples were adjusted to a volume of 100 mL with deionized water to carry out the analysis of N, P, K, and Pb. The protein nitrogen was estimated by applying the modified micro-Kjeldahl method as described by Peach and Tracey^56^.

Phosphorous concentration was determined by the colorimetric method^57^, and the concentrations of potassium (K) were estimated by flame photometry. Nitrogen, phosphorous, and potassium were expressed as gram/100 g of the plants’ parts dry weight, while Pb^2+^ was expressed as μg/g of the extract’s dry weight.

The method of Bates et al.^58^ was used for the proline contents determination (mg/g f.wt.). The total carbohydrates (mg/g dry wt.) were established according to the assay of Dubois et al.^46^**.** Activities of the catalase and peroxidase enzymes were assayed in fresh leaves’ tissue extracts according to Chance and Maehly^59^, with slight modification**.** One unit of catalase activity was specified as the amount of enzyme that reduced 50% of the H_2_O_2_ in 60 s at 25 °C and was expressed as µg/g/ f. wt., while one unit of peroxidase activity was defined as the amount of enzyme that catalyzed the conversion of one micromole of H_2_O_2_ per minute at 25 °C and were expressed as µg/g/ f. wt.,^60^. The lipid peroxidation level was estimated by assessing the levels of malondialdehyde (MDA) by the modification method of Zhou^61^ and the results were expressed as nmol/g dry wt**.**

### Statistical analysis

The impact of lead stress on wheat plants utilized a Randomized Complete Block Design with 3 blocks. Prior to analysis, we ensured the fulfillment of key assumptions, including normal distribution and homogeneity of variance in the error terms, by scrutinizing residuals, following the methodology outlined by Snedecor and Cochran’s^62^. The independence assumption was upheld as treatments were randomized within each block.

Upon detecting significant treatment effects, we conducted multiple means comparisons using the Duncan range test (DMRT) method at a 5% significance level to generate letter groupings indicating significant differences between treatments. This post-hoc analysis allowed for a nuanced understanding of treatment effects on wheat plants under lead stress conditions. The statistical analysis was executed using the General Linear Model (GLM) procedure of **SAS**^63^, providing a robust framework for evaluating treatment effects and facilitating post-hoc comparisons. This comprehensive approach ensured an accurate assessment of the impact of lead stress and the Bio-Stimulating Effect on wheat plants. Means followed by the same letters are not significantly different at *p* < 0.05.

### Experimental research and field studies on plants

The collection of wheat plants was with relevant institutional, national, and international guidelines and legislation.

## Results

### Isolation and identification of endophytic fungi

**Figure 1:**
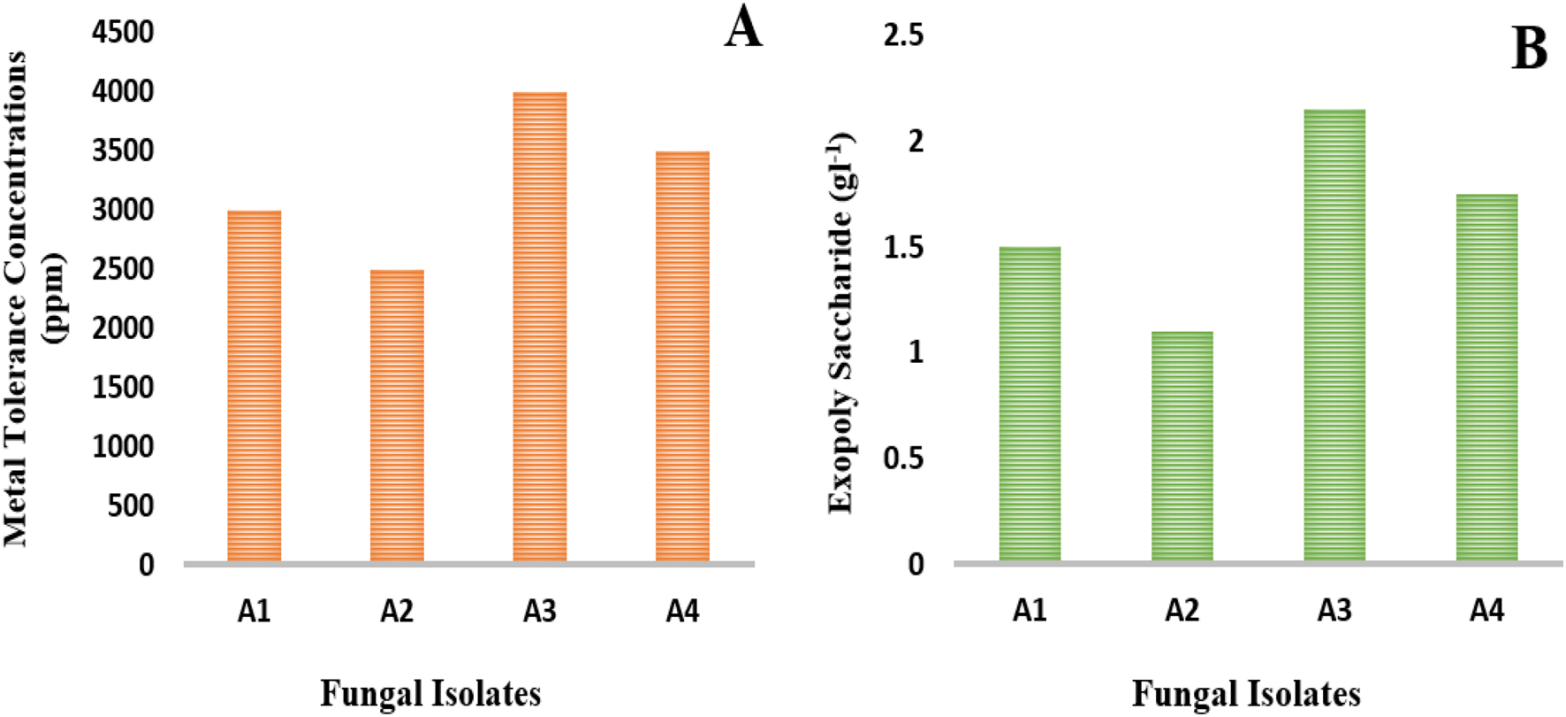
(**A**): The metal tolerance ability of isolated endophytic fungi, (**B**): EPS production by isolated endophytic fungi.

In the present study, four different endophytic fungal isolates with different morphological colonies were isolated from wheat plants in Wadi El-Natroon. Based on morphological characteristics under a light microscope, three isolates (A1, A2, and A3) were identified as species of *Aspergillus*, while (A4) was identified as *Penicillium* sp. as shown in Table 1. Additionally, all of them showed varying levels of Pb^2+^ tolerance (Fig. 1A). The fungal isolate *Aspergillus sp.* A3 was found to exhibit the highest MTC value (4000 ppm) for Pb^2+^ followed by *Penicillium sp* A4 (3500 ppm), *Aspergillus sp* A1 (3000 ppm), and *Aspergillus sp* A2 (2500 ppm), respectively. The fungal isolate *Aspergillus sp.* A3 was selected as the most tolerant one. Next, the four fungal isolates were screened for EPS production. The results of screening indicated that the fungal isolate *Aspergillus sp. A3* was the most promising EPS producer (2.15 gl^−1^) followed by *Penicillium sp* A4 (1.75 gl^−1^), *Aspergillus sp* A2 (1.50 gl^−1^), and *Aspergillus sp* A1 (1.10 gl^−1^), respectively (Fig. 1B).

**Table 1:** Cultural characteristics of endophytic fungal isolates on PDA medium and their microscopic examination under the light microscope (200×).

Isolate code	Identification	Culture	Microscopic examination
(A1)	*Aspergillus* sp.		
(A2)	*Aspergillus* sp.		
(A3)	*Aspergillus* sp.		
(A4)	*Penicillium* sp.

### Identification of the more lead-tolerable and EPS producer *Aspergillus sp.* A3

**Figure 2:**
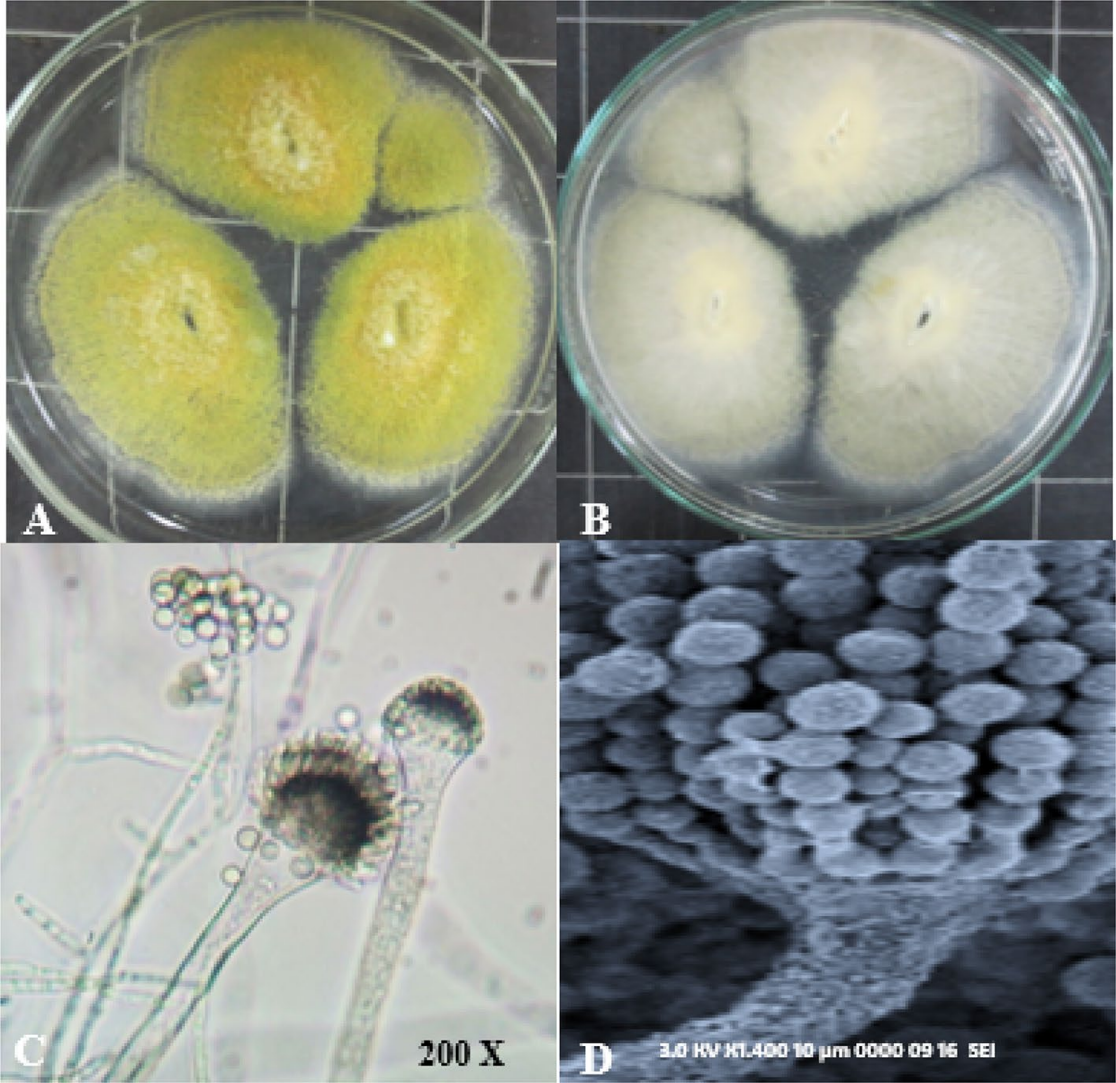
(**A**–**D**): The morphological features of endophytic *Aspergillus sp. A3,* (**A** = front and **B** = reverse) later 7 days incubation on PDA media, (**C**): microscopic characteristics of fungus conidiophores and conidia (magnification power = 200). and (**D**): Scanning electron microscope explaining the conidiophore and conidia (1400×).

The endophytic *Aspergillus sp.* A3 shows a rapid growth rate reaching 38mm diameter after 7 days of incubation at 28 ± 2 °C on PDA. The colonies with green olivaceous surfaces, with reverse pale white background (Fig. 2A and B). The microscopic examination revealed a radiating conidial head while the conidiophores appeared heavy-walled, uncolored, and coarsely roughened. Sterigmata were arranged in a single series, conidia appeared as sub-globose and round (Fig. 2C and D).

### Molecular identification

**Figure 3:**
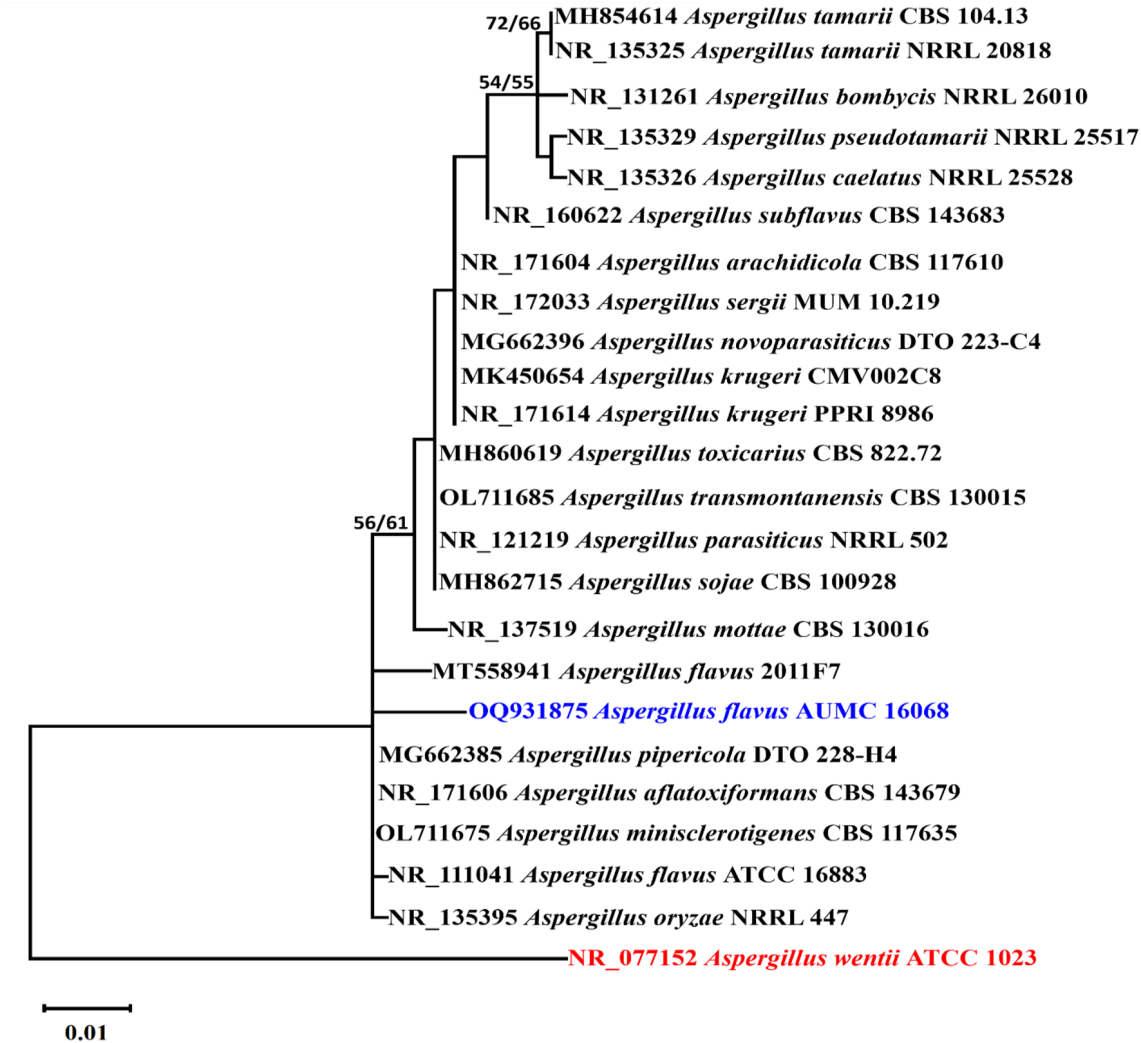
Evolutionary tree derived from a computational searching (1000 replications) of endophytic *Aspergillus flavus* AUMC 16,068 (blue color) in comparison to other *Aspergillus*: section Flavi ITS sequences found in GenBank. Bootstrap support values are displayed above/below the corresponding nodes for ML/MP when they are equal to or more than 50%. The tree is rooted to *Aspergillus wentii* ATCC 1023 as an outgroup (in red color).

Referring to a mega blast query of NCBIs GenBank nucleotide record, the most similar hits using ITS sequence of *Aspergillus* sp. AUMC 16,068 are *Aspergillus flavus* 2011F7 [GenBank accession numberMT558941; identities = 578/580 (99.66%); Gaps = 1/580 (0%)]. In the current study, *Aspergillus* sp. AUMC 16,068 was compared to other *Aspergillus* species related to section Flavi utilizing a phylogenetic assessment constructed on ITS sequencing information. 24 sequences totaling 634 characters were created from the ITS data set, of which 524 could be effectively aligned (with no gaps or N), 49 characters were rated as a variable, and 8 were rated as informative. The Maximum Parsimony method was used to create 8 trees to deduce evolutionary history. The most parsimonious phylogenetic tree for all sites and parsimony-informative sites is shown in Fig. 1 with a tree distance of 77 steps, a maximum log-likelihood of (− 1253.29), uniformity index of 0.695652, retention index of 0.895522, and composite index of 0.622972. As shown in Fig. 3, the phylogenetic tree, *Aspergillus* sp. AUMC 16,068 was clustered with *Aspergillus* section Flavi. It is located near the strain *A. flavus* 2011F7. As a result, this strain is identified here as *Aspergillus flavus*. ITS sequence was uploaded to GenBank as OQ931875.

### Characterization of EPS

#### Evaluation of the total sugar content of EPS

In the current investigation, the results of the chemical composition of EPS produced by endophytic *Aspergillus flavus* AUMC 16,068 evoked the presence of carbohydrates.

#### FT-IR spectrum

**Figure 4:**
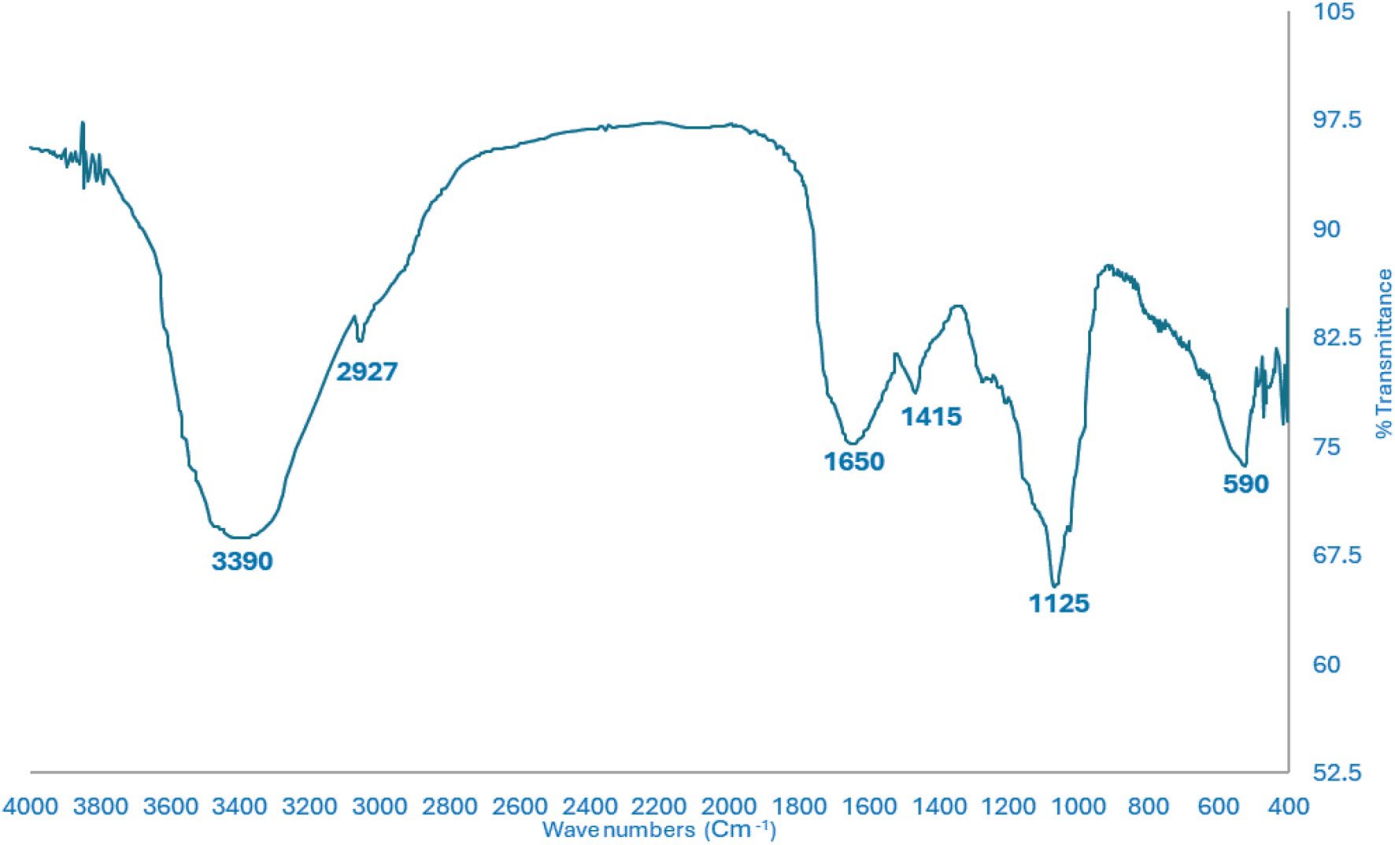
FTIR Spectrum of EPS produced by endophytic *Aspergillus flavus* AUMC 16,068.

The FT-IR spectrum of the EPS displayed numerous different and sharp absorptions as shown in (Fig. 4). The presence of a polyhydroxilic compound is shown by the absorption band at 3390 cm^−1^ on the stretching vibration of the hydroxyl groups of linkages (OH). The C–H band exhibits strong, stretching vibrations, as indicated by the peak at 2927 cm^−1^. Additionally, bands between 1415 and 1653 cm^−1^ were indicative of the amide I –CONH– group’s vibration in proteins. A wide stretching of C–O–C, and C–O correlated with polysaccharides at 900–1200 cm^−1^. The peaks in the 1000–1125 cm^−1^ range correspond to the linkage bond properties of uronic acid and O–acetyl ester.

### Soil characterization

The used soil was sandy loam with slightly acidic to neutral pH (6.91), electrical conductivity (1662 μS cm^**−**1^), organic carbon (2.24%), and organic matter contents (3.8%) as shown in Table 2.

**Table 2:** Physical and chemical properties of soil.

Physical properties	Particle size (%)	Total sand	68.25
		Silt	11.50
		Clay	20.25
	Texture class		Sandy loam soil
Chemical properties	pH		6.91
	EC mm hos/cm		0.82
	Soluble cations meq/L	Ca^++^	12.76
		Mg^++^	7.23
		Na^+^	9.74
		K^+^	3.78
	Soluble anions meq/L	CO_3_^-^	0.00
		HCO_3_	2.11
		Cl^−^	3.74
		SO_4_^− −^	0.43
	Organic carbon %	2.24	
	Organic matter %	3.80	
Heavy metal ion con. µg/g	Pb^2+^	0.004	

### Effect of spore inoculation of endophytic *Aspergillus flavus*AUMC 16,068 and its EPS on some growth parameters of wheat plants exposed to lead contamination

**Figure 5:**
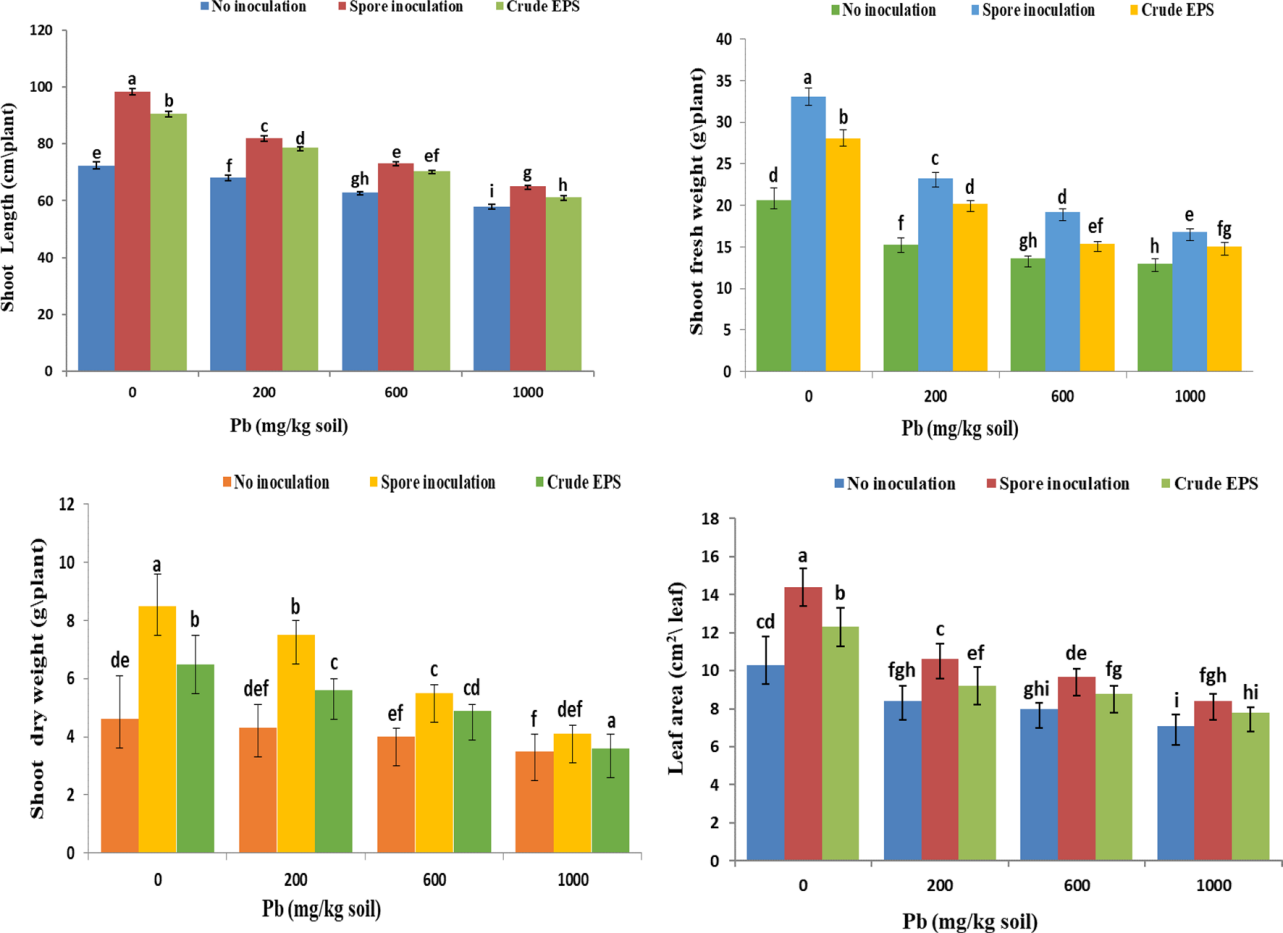
Effect of spore inoculation of endophytic *Aspergillus flavus* AUMC 16,068 and its EPS on growth parameters of wheat plants grown under lead contamination.

In the current study, the presented outcomes in Fig. 5 showed the effect of different concentrations of Pb as well as the effect of wheat grain inoculated with *A. flavus spores* and its EPS on some growth parameters of wheat plants. Generally, all growth parameters decreased with increasing Pb^**2+**^ concentrations. The highest Pb^**2+**^ concentration (1000 mg/kg soil) reduced shoot length, shoot fresh, and dry weights, and leaf area by 19.7%, 36.9%, 23.9%, and 31.1%, respectively compared to the control one. This drastic effect of lead ions improved with inoculation by fungal spore and EPS respectively at all Pb^**2+**^ concentrations. The highest increment value was in plants grown under low Pb concentration (200 ppm). It is interesting that the inoculation of wheat cultivar grain with*. flavus* spores, showed a significant enhancement in all growth parameters of wheat, increasing the shoot lengths (20%), shoot fresh weight (50%), shoot dry weight (74.4%), and leaves area (26%), compared with lead-contaminated uninoculated cultivars. concerning the effect of crude EPS on the treated plants with 200ppm Pb, it was noticed that pretreatment of grains with crude EPS gave a significant increase in the shoot lengths (14.4%), shoot fresh weight (32%), shoot dry weight (30.2%) and as well as leaves area (9.5%) compared to contaminated untreated wheat plants. In this investigation, we decided to select endophytic fungi that could oppose lead toxicity in wheat plants. At the same time, the isolated fungi produce exopolysaccharides that help increase nutrient uptake and promote plant growth of wheat plants in soils of heavy environmental stress**.**

### Effect of spore inoculation of endophytic *Aspergillus flavus* AUMC 16,068 and its EPS on antioxidant enzymes at the heading stage of wheat plants grown under lead stress

**Figure 6:**
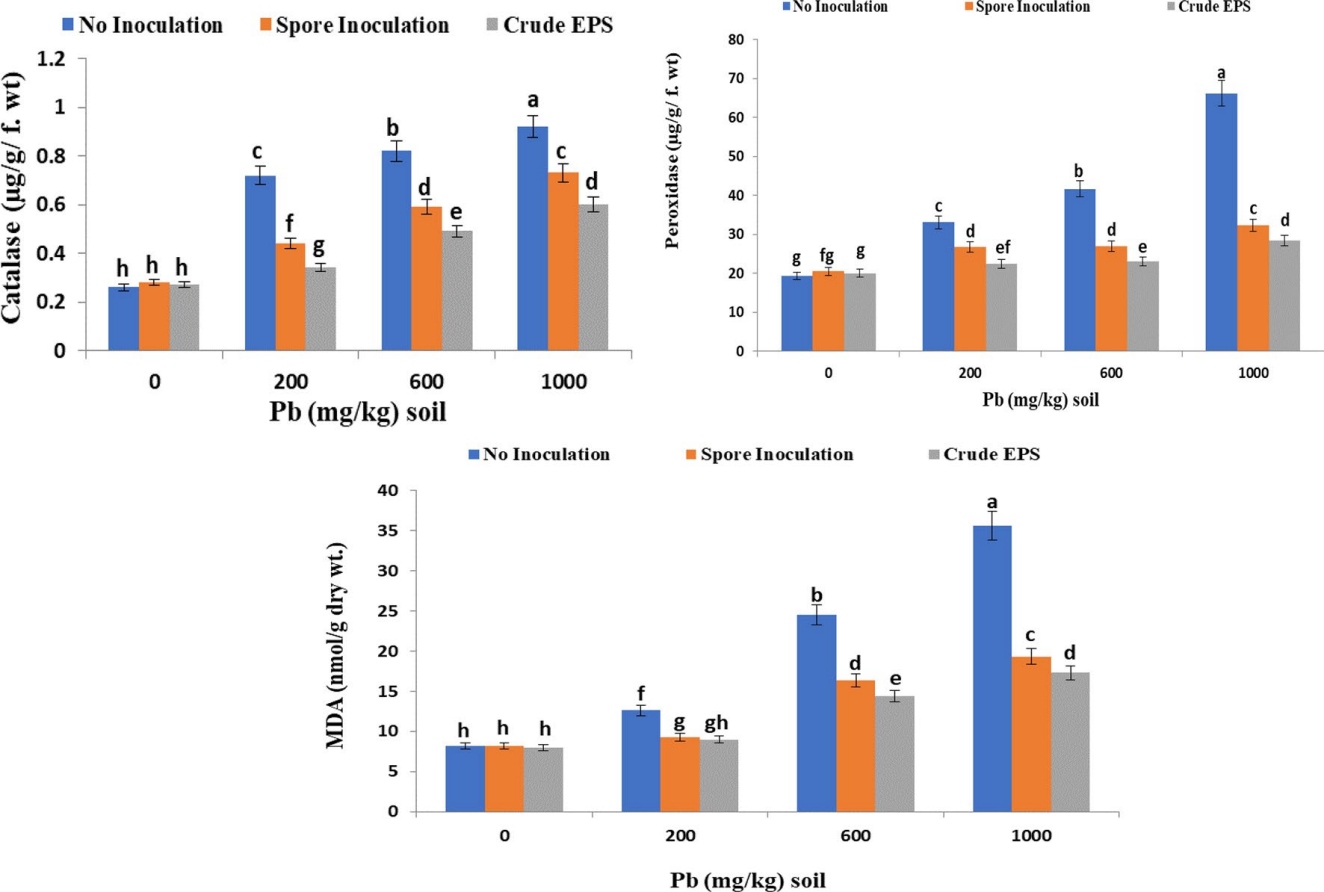
Effect of spore inoculation of endophytic *Aspergillus flavus* AUMC 16,068 and its EPS on antioxidant enzymes and (MDA) at the heading of wheat plants grown under lead stress.

The data gained in the current work confirmed that there was a significant increase in catalase (CAT), peroxidase (POD) activity as well as lipid peroxidation (MDA) with increasing Pb concentrations in both inoculated and non-inoculated wheat plants (Fig. 6). Increasing Pb^2+^ concentrations in the soil significantly increase MDA & CAT and POD activity in plant leaves compared to control (untreated non-contaminated plants). Moreover, inoculation with endophytic *Aspergillus flavus* AUMC 16,068 spores causes a reduction in the levels of stress signs (CAT, POD, and MDA) in wheat plants. The grains treatment with EPS was decent handling, as it caused a significant decline in (CAT, POD, and MDA) levels.

### Effect of spore inoculation of endophytic *Aspergillus flavus* AUMC 16,068 and its EPS on carbohydrate, protein, and proline contents of wheat plants under lead stress

**Figure 7:**
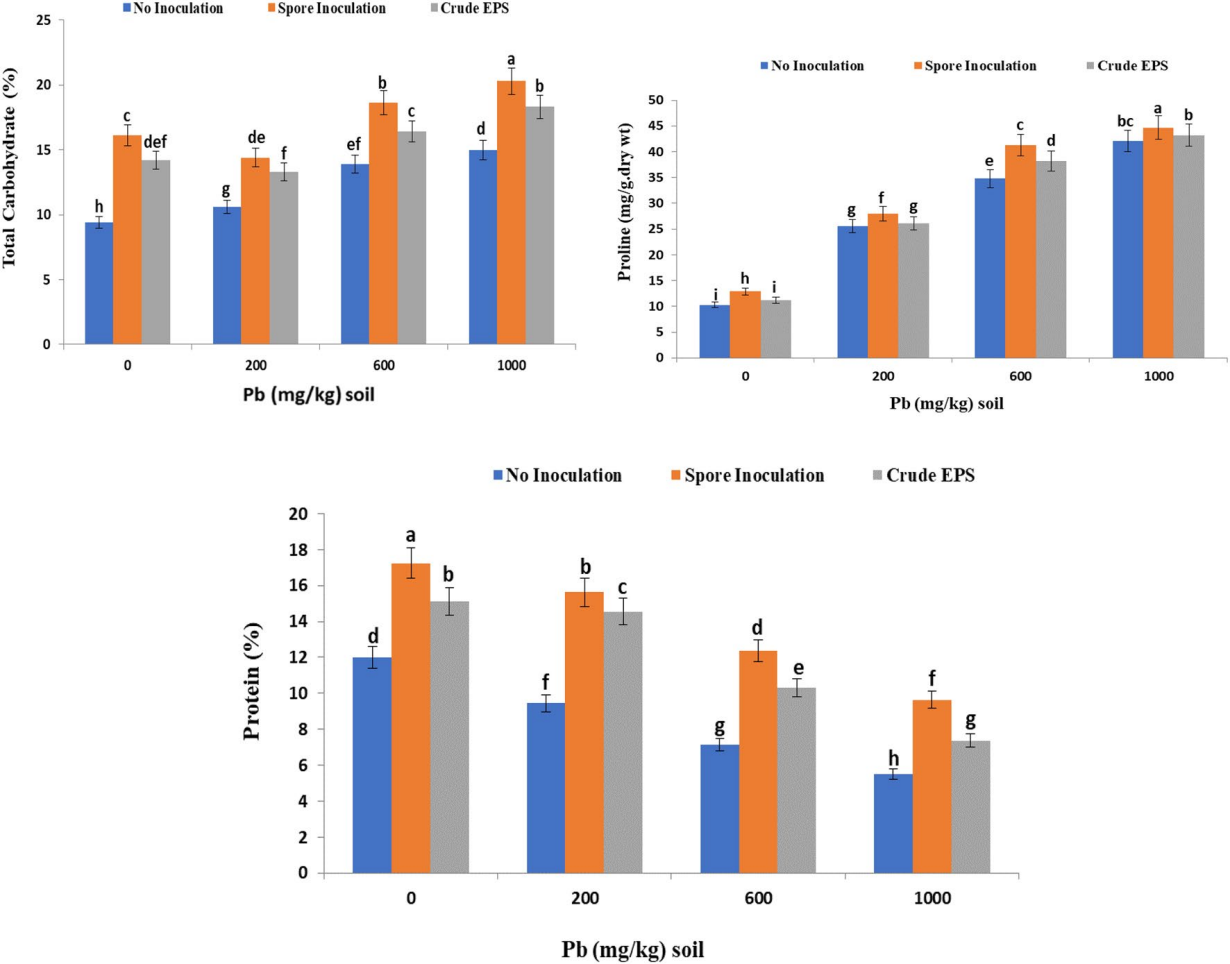
Effect of spore inoculation of endophytic *Aspergillus flavus* AUMC 16,068 and its EPS on total carbohydrates, proline, and protein contents at the heading stage of wheat plants under lead stress.

Regarding carbohydrate contents of the stressed wheat plant, the results revealed a highly significant increase in carbohydrate contents of stressed wheat plants compared to the control one, especially at a high lead concentration (1000 ppm). The formation and synthesis of diverse suitable solutes and osmolytes are recognized as an important defensive mechanism for wheat plants cultivated under heavy metals stress conditions (Fig. 7).

Additionally, our results also demonstrated that total carbohydrates increased significantly in shoots of wheat plants exposed to different lead concentrations and inoculated with *Aspergillus flavus* AUMC 16068spores. The increment reached 28% in inoculated plants grown under 200 ppm compared to non-inoculated plants (Fig. 7). It was noticed that inoculation with crude EPS increases the content of carbohydrates by (25%) in plants grown under the same pb concentration.

Furthermore, the outcomes recorded in Fig. 7 revealed that lead contamination manifests a rise in the free proline contents in the shoot of the wheat plant. The concentration of proline was significantly increased in both inoculated and non-inoculated plants under lead toxicity. It is attractive that inoculation with *A. flavus* spores boosted the resistance of the plant and showed substantial increases in free proline contents by (18.6%) in wheat plants grown under lead concentration (600 mg). Additionally, inoculation with crude EPS showed considerable increases in free proline contents by (9.7%) in plants at 600 mg lead concentration.

Interestingly, the results evoked that protein content decreased significantly in shoots of stressed wheat plants compared to control one (uncontaminated plants) due to heavy metal application. Also, there is a progressive increment of protein concentration in plants grown under lead contamination and inoculated with *Aspergillus flavus* AUMC 16,068 spore compared to contaminated untreated plants. On the other hand, the treatment of wheat plants with crude EPS proved substantial enhancement in protein content when compared to contaminated untreated plants (Fig. 7).

**Figure 8:**
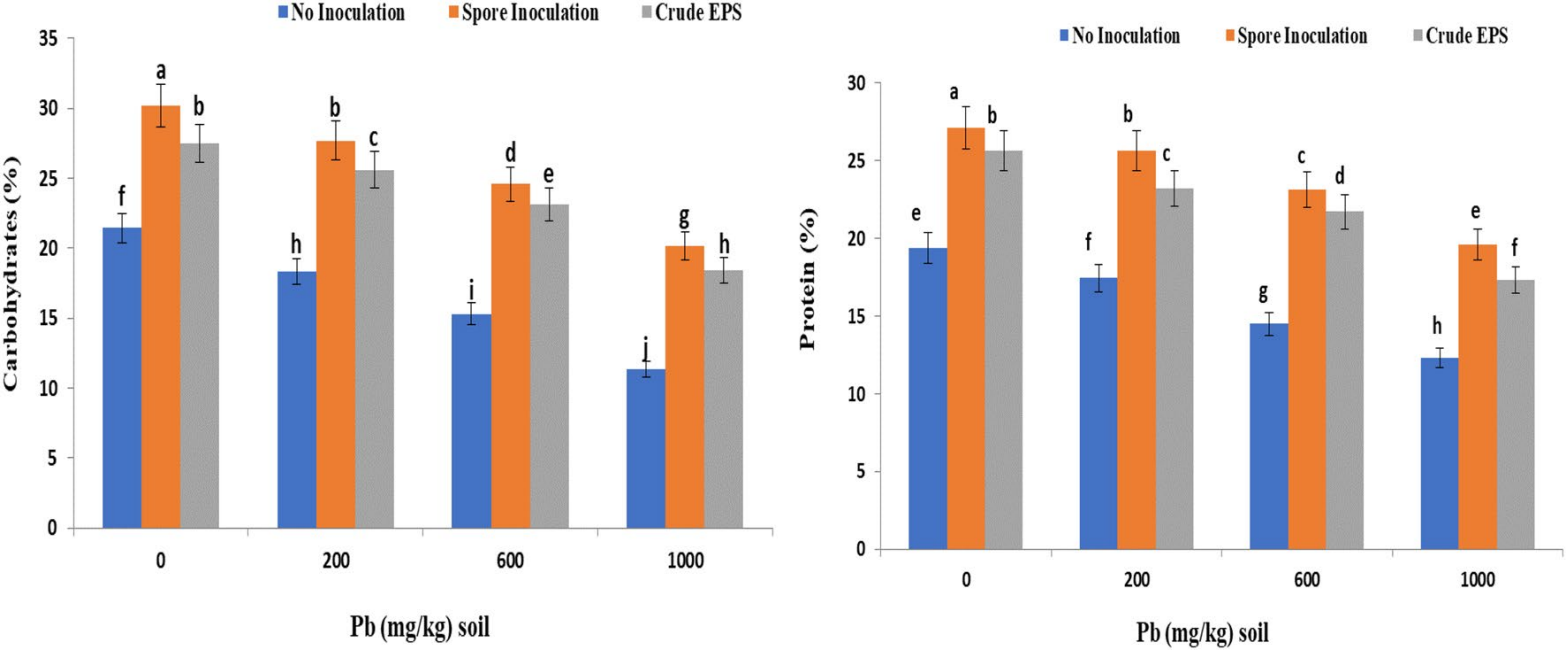
Effect of spore inoculation of endophytic *Aspergillus flavus* AUMC 16,068 and its EPS on total carbohydrates and protein contents of wheat grains under lead stress.

The results illustrated in Fig. 8 demonstrated the effect of lead treatments as well as the application of endophytic *Aspergillus flavus* AUMC 16,068 and its EPS on the total carbohydrates and total proteins of grains in wheat plants. Data showed that significant and pronounced depressive effect of lead on carbohydrates and proteins at all lead concentrations. The plants grown under high lead concentration (1000 ppm) showed a most significant decrease in total carbohydrates content as well as total proteins (47% and 36%) respectively, in grains compared to uncontaminated ones. In this connection**,** data also reveals that the treatments with the fungal spores and its EPS resulted in an increase of total carbohydrates and proteins in grains of lead-stressed plants compared to contaminated untreated plants**.**

### Effect of spores inoculation of endophytic *Aspergillus flavus* AUMC 16,068 and its EPS on the minerals content of wheat plants under lead toxicity

**Figure 9:**
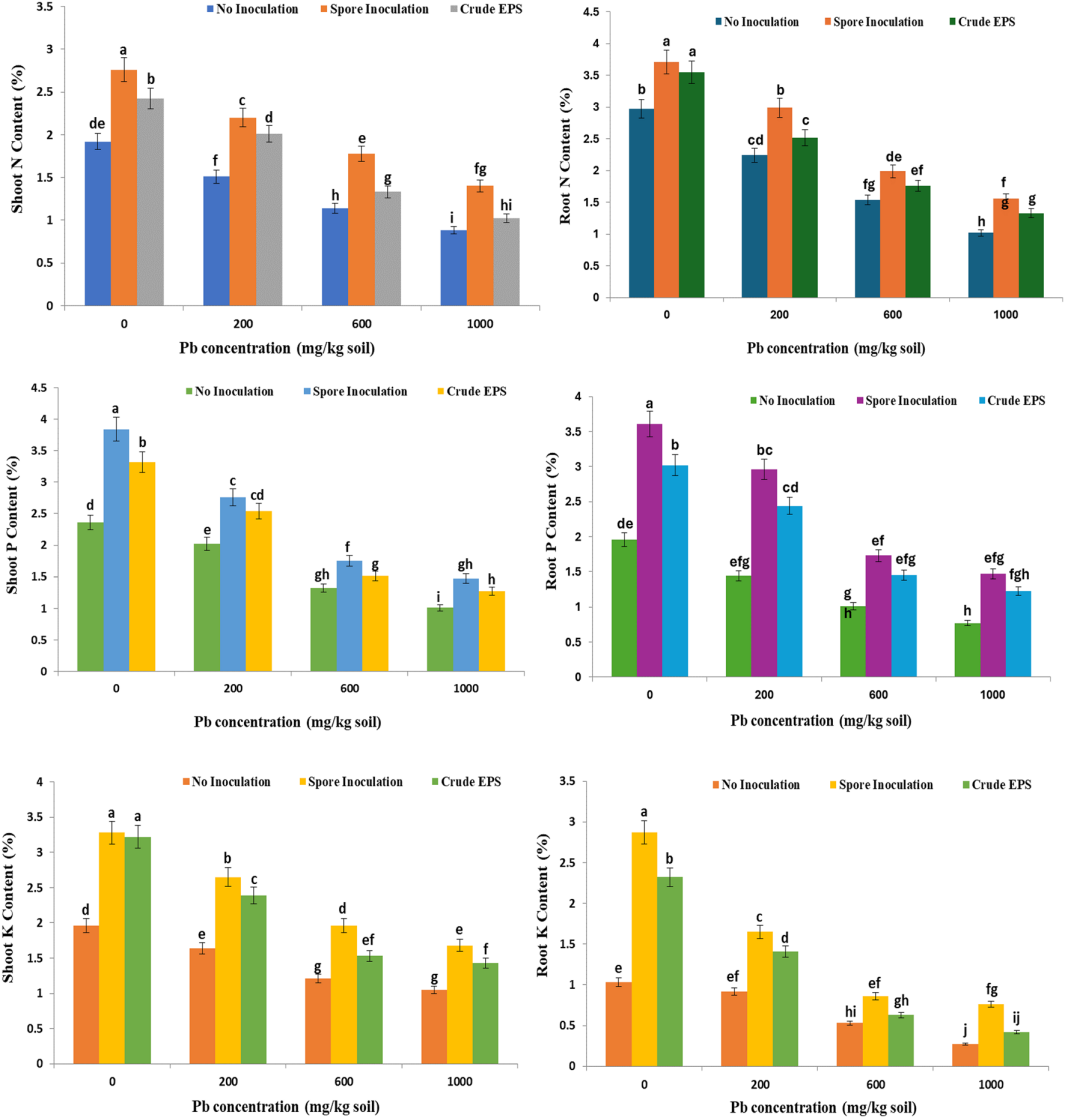
Effect of spore inoculation of endophytic *Aspergillus flavus* AUMC 16,068 and its EPS on minerals content of wheat plants under lead toxicity.

The effect of lead contamination on the uptake of macronutrients in shoots and roots of wheat plants was illustrated in Fig. 9. The results evoked a significant reduction of N, P, and K uptake with increasing lead concentrations in the soil, especially the plants grown under high lead concentrations (1000 mg). Also, the results disclosed that the inoculation of wheat cultivar grains with *A. flavus* spores significantly increased all macronutrient content in shoots and roots under different lead concentrations compared to contaminated non-inoculated plants.

### Effect of spores inoculation of endophytic *Aspergillus flavus* AUMC 16,068 and its EPS contents on Pb accumulation in the root, shoot, and grains of wheat plants

**Figure 10:**
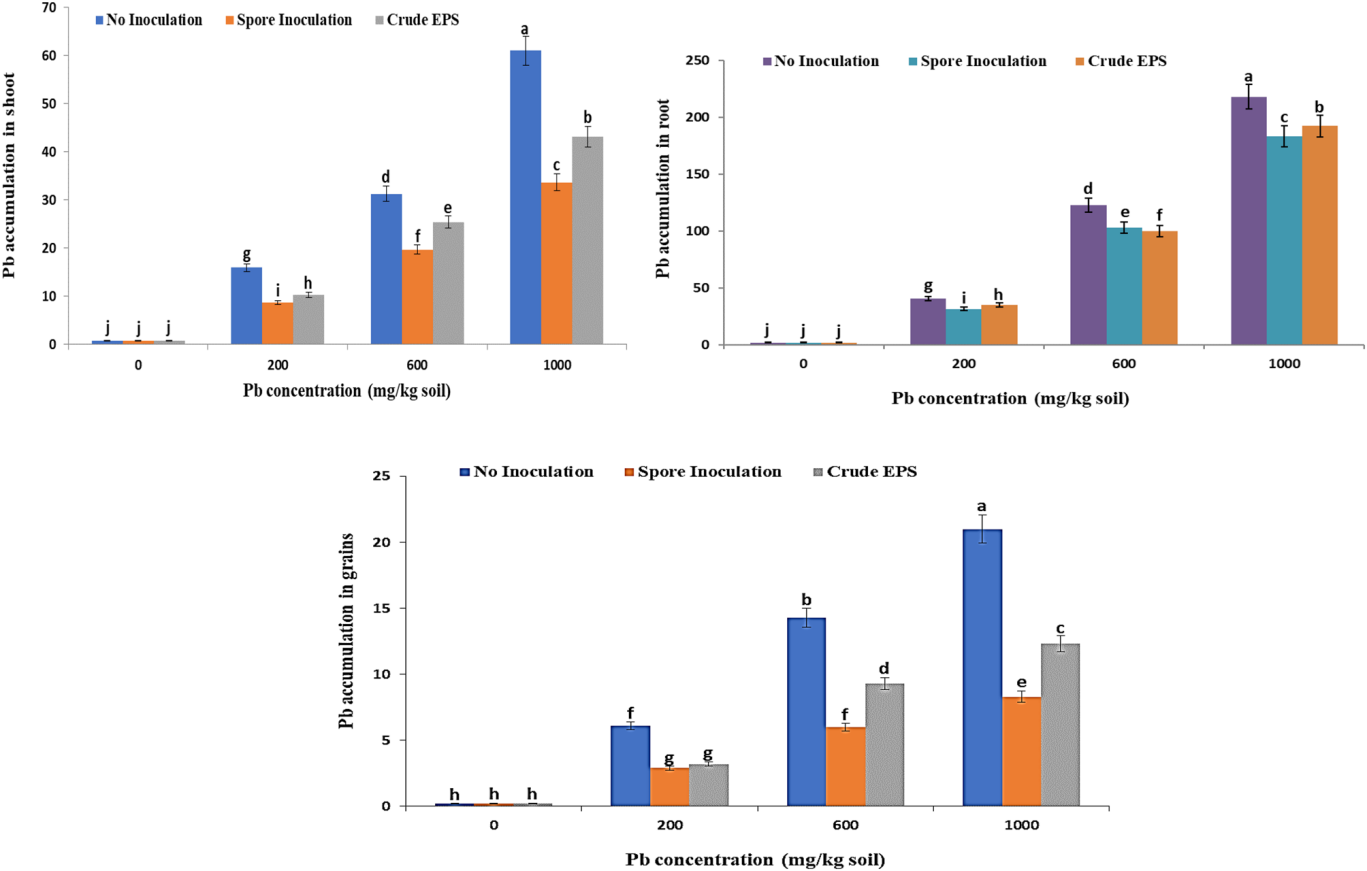
Effect of spore inoculation of endophytic *Aspergillus flavus*AUMC 16,068 and its EPS on Pb accumulation in shoot, root, and grains of wheat plants.

Lead accumulation in roots, shoots, and grains of wheat plants increased with increasing external metal concentrations. Roots accumulated more metal as compared to shoots and grains. The highest lead accumulation was observed in root, shoots, and grains, respectively in plants grown at 1000 mg kg^−1^ Pb^**2+**^ stress while decreased to (183.3, 33.7 &8.3 µg/g d wt., respectively in root, shoot, and grain upon spore inoculated treatment (Fig. 10).

Additionally, by the inoculation of wheat cultivar with endophytic *Aspergillus flavus* AUMC 16,068 spores and crude EPS, it was noticed that fungal inoculation significantly decreased the Pb uptake in each concentration followed by application of crude EPS respectively as compared to their respective non-inoculated wheat plant. The current investigation also proved that inoculation with endophytic lead tolerant *A. flavus AUMC* 16,068 and its respective EPS improved Pb accumulation in root, shoot, and grains of wheat plants in comparison to plants grown under Pb stress deprived of inoculation.

## Discussion

The rising concerns of the increasing global population, limiting arable land, and the intensifying hazards posed by the effects of climate change place a premium on developing novel techniques and strategies for increasing yield potential under stressful conditions. Plant stress is caused by various biotic and abiotic variables, including drought, temperature, salinity, and heavy metals, which cause significant changes in plants. Abiotic stresses are one of the most important factors affecting crop yield and productivity in the agriculture sector. These stresses allow the formation of reactive oxygen species, the destruction of membranes, and plant metabolic activity^64^. Phytoremediation is a sustainable strategy that uses plants’ metal tolerance capability to reclaim heavy metal damaged areas^65,66^.

Endophytic fungi are a prospective source of natural fertilizers, growth regulators, and natural antimicrobials that can be employed as green alternatives to agrochemicals. Among the main producers of active metabolites gained from fungi is *Aspergillus*^67^. Moreover, Barton & d’Errico^68^ revealed that *Aspergillus* fungus has beneficial effects on plants and is used in several ways and treatments to maintain, encourage, and improve plant growth. Numerous studies have suggested that endophytic fungi could potentially be used to produce plant growth hormones (GA and IAA), such as *Phoma* sp. GAH7 isolated from cucumber roots yielded high levels of GA3, GA4, GA9, and GA19, which has been employed as a control for GA biosynthesis^69^. Moreover, endophytic *Paecilomyce svariotii* from the roots of cucumbers produces high amounts of IAA and Gas^70^. Endophytic microorganisms are promising resources of biofertilizers, natural antimicrobial compounds, and growth promotors, which can be used as green advances to reduce the use of agrochemicals^71,72^. Other researchers have confirmed similar findings, demonstrating the beneficial effects of endophytic microorganisms where it is important for developing feasible replacements for chemicals like fungicides and insecticides, as well as the recurrent problems of their uncritical use and could apply the endophytes for plant growth promotion and prevention of fungal infections^73,74^**.** The plant growth-promoting abilities of endophytic fungus, and recent advances in our knowledge of some of the mechanisms, imply that this interesting source warrants additional exploration for possible future use in agriculture. Many researchers indicated that some important products that can help the native microbial strains tolerate and degrade heavy metals are exopolysaccharides (EPS), biosurfactants, and peroxidase enzymes. EPSs provide several biological functions including the defense against environmental stress factors and the interactions with other organisms^75^. According to Asadi et al.^76^, the fungal EPSs differ in terms of monosaccharide linkage arrangements, entities of monosaccharide, molecular weight, branching extent, glycosidic bonding, and configuration. Many fungi strains have been identified so far that can generate useful EPSs. Despite the advantages of these functional EPSs, there have only been a few investigations on their commercial application^77^. The yield of microbial EPSs differs depending on the type of strain and parameters including the fermentation’s pH, temperature, etc.^78,79^. Although bacteria and fungi are the utmost frequent resources of microbial EPSs^80^, fungal EPSs have got less attention ^81^. The biological activity of EPS extracts results from their composition^82,83^. In contrast to marine or plant-based PSs, microbial EPSs can be produced in just a few days, don’t compete with arable lands, can be supplied with agro-industrial waste, and are often easy to obtain and purify^78^**,** their standard production yields range from 0.0022 to 100 g/L^84^. Under simple production conditions, certain fungal species, such as *Aureobasidium pullulans*, can produce 40 g/L EPS^85^.

The chemical constituents of EPS of endophytic *Aspergillus flavus* AUMC 16,068 evoked the presence of carbohydrates. Characteristic bands from the FTIR could be connected to various functional groups (Fig. 4). Our results were consistent with the earlier reports of^86^.

Amongst soil characteristics, soil pH has an immense impact on metal diversification and solubility in both soil and soil solution^89^, however, in addition to heavy metal retention, the amount of organic matter has a significant effect on cation and buffer capacity. Metals in organic-rich soils polluted with heavy metals are thus less bioavailable and mobile than metals in mineral soils^90^. Lead is an immobile metal in soil because it rapidly forms a precipitate with limited water solubility within the soil matrix, making it in many circumstances unavailable. According to soil qualities, such factors may act alone or in combination, altering the soil properties of the lead available as well as the rate of uptake by plants^91^.

Generally, all growth parameters of wheat plants under study were decreased with increasing Pb^**2+**^ concentrations (Fig. 5). Numerous fungi’s impacts on plants have also been thoroughly studied, with a stronger emphasis on promoting plant growth^92^. The same outcomes were reported by Rachidi et al.^93^, who found that inoculating plants with growth-promoting microbes improved physiological features like root and shoot length, fresh and dry weight, leaf surface area, as well as biochemical attributes like protein and proline content, and antioxidant enzymes activity in the tomato plants grown under stress**.** On the other hand, the current results were in line with many other previous reports stating the role of EPS inoculations in improving plant growth^94,95^. Moreover, Khan & Bano^96^ revealed that the treatment with EPS increased the shoot of wheat grown under rainfed conditions. Additionally, under drought stress conditions, inoculation of maize seeds with EPS-producing bacterial strains and their corresponding EPS-enhanced plant biomass, leaf area, both shoot and root length, and leaf carbohydrate and protein levels^97^. Also, Rachidi et al.^93^ noted that the treatment with 1 mg mL of crude EPS resulted in a substantial increase in shoot size, node counts, root dry weight (RDW), and shoot dry weight measurements. Polysaccharides from seaweed extracts^98^ and halophyte plant *Lobularia maritima*^99^, improved growth and development in wheat.

Heavy metals’ inhibitory effects differed according to plant age, exposure length, heavy metal content, and type^100,101^. Besides, Nagarathnamma et al.^102^ showed that the decreasing pattern in plant development could be caused by the inhibition of metabolic activities that are associated with regular plant growth. Also, Gopal & Khurana^103^ stated that the excess of different heavy metals such as Cd and Pb resulted in stunted growth and reduced biomass accumulation. Additionally, Ashraf et al.^104^ revealed that metal contamination restricted seedling growth of four wheat (*Triticum aestivum *L.) cultivars because interactions with enzymes and biochemical reactions occur inside the plant body. High heavy metal concentrations inhibited the translocation of soluble substances and the accumulation of dry matter in pea plants^105^. The decrease in the length of plant height was found to be correlated with a decrease of the endogenous auxin which was particularly observed under high Cd and Pb metals, and heavy metals can release ethylene within the plants leading to an inhibition of auxin transports and depression of plant height^106^.

According to the data gained in the current work, there was a significant increase in catalase (CAT), peroxidase (POD) activity as well as lipid peroxidation (MDA) with increasing Pb concentrations in both inoculated and non-inoculated wheat plants (Fig. 6). Our results were in agreement with Panda^107^ who disclosed that lead stress-induced oxidative stress in plants. Under stress, reactive oxygen species (ROS) are routinely formed and have high oxidizing capabilities that can target many sorts of biomolecules. Also, Del Rio et al.^108^ indicated that the excessive production of ROS by Pb^2+^ stress damages polyunsaturated lipids in cell membranes, resulting in the formation of MDA, and this is used as a biomarker to assess the intensity and scale of oxidative stress. Ex-polysaccharide promotes microbe-microbe and microbe-plant interaction, afford antioxidants, provide nutrients, and store carbon to assist plant growth^109^**.**

The results revealed a highly significant increase in carbohydrate contents of stressed wheat plants compared to the control one. On the other hand, the protein content decreased significantly in shoots of stressed wheat plants compared to control one (uncontaminated plants) due to heavy metal application (Fig. 7 and 8). These findings were comparable with the findings of Rachidi et al.^93^, who found that the microalgae EPS treatment had a substantial effect on chlorophyll and protein content, as well as NAD-GDH and NR enzymatic activities in tomato plant leaves when compared to the control. Plant development following EPS treatment was accompanied by an increase in its essential elements such as nitrogen, enzyme activity, and protein level. Similar investigations found that carboxylated or sulfated polysaccharides improved photosynthesis, nitrate absorption, and fundamental metabolic processes^110,111^**.** Also**,** Qurashi and Sabri^94^ reported a maximum increase in the total soluble sugar content and protein content of chickpea plants upon bacterial exopolysaccharide inoculation. The accumulation of carbohydrates under metal stress protects plant cells by vacuolar modification and balancing the cytosol^112^. Moreover, the increase in carbohydrate content induced by Pb and Cd may suggest that flax and canola plants operate a metabolic mechanism for channeling heavy metals within their cells. This increment of carbohydrates may be due to the inhibitory effect of such metals on phloem translocation^113^. These effects related to this study were consistent with that of^93^ who informed that the microalgae EPS treatment of plants had a significant effect on protein content. Lead (Pb) diminished soluble protein in the roots and leaves of cotton plants^114^. Besides, Awan, et al.^115^ conveyed a decline in the amount of proteins in rice plants and this may be due to increased protein degradation produced by increased protease activity revealed under Pb stress conditions. Lead can induce protein disintegration due to the harmful effects of reactive oxygen species, resulting in lower protein content. In this link, El-Sayed et al.^116^ stated that metal stress (Pb) can act at multiple sites to block numerous enzymes bearing functioning sulfhydryl groups. It harms normal protein synthesis in bean plants by altering the pathways and protein synthesis.

The outcomes of this work revealed that lead contamination manifests a rise in the free proline contents in the shoot of the wheat plant. Proline improves plant stress tolerance through different mechanisms such as osmoregulation, enzyme denaturation prevention, and protein synthesis stability^117^. Proline accumulation in plants in response to heavy metal exposure appears to be common^118^. Moreover, in responses to exposure to heavy metals, proline accumulation has been noted in the leaves of various plants, which could be due to a decrease in proline breakdown and/or an increase in proline synthesis, as well as the breakdown of protein resulting in liberated amino acids^66,119,120^.

The excessive accumulation and production of many compatible solutes and osmolytes is a critical defensive mechanism for plants cultivated under metal stress conditions. There is a favorable relationship between proline accumulation and plants being subjected to various stressors. Proline improves plant stress tolerance by preserving cell turgor or osmotic equilibrium, protecting membranes, and imposing the concentration of reactive oxygen species (ROS) within typical level ^121^. As a result, plants are protected against oxidative damage^7,122,123^. A study conducted by Naseem et al.^124^ indicated that Plants inoculated with EPS-producing bacteria showed a higher accumulation of proline, sugars, and free amino acids under water deficit stress. Also, Isfahani et al.^125^ recorded that the proline content was significantly decreased in tomatoes under salinity stress by the use of EPS, and bacterial treatments.

A significant reduction of N, P, and K uptake with increasing lead concentrations in the soil was recorded in our results and the inoculation of wheat cultivar grains with *A. flavus* spores significantly increased all macronutrient content in shoots and roots under different lead concentrations compared to contaminated non-inoculated plants (Fig. 9). The results were in the same line with the study of Rouphael et al.^126^ who observed an improvement in nutrient uptake when the endophytic fungus *Glomus intraradices* G72 was used. The improved nutritional status in the open field experiments (N, P, K, Na, and Mg) recorded in wheat plants coming from the inoculated grains may be considered as an indirect effect of the beneficial microorganisms. Also, Kevresan et al.^127^ reported a decrease in nitrate and protein metabolism of young pea plants due to lead contamination. Lead may attach to sulfhydryl groups in proteins, which could damage protein structure or decrease action, causing toxicity^128^. The decline in carbohydrate levels because of lead contamination may be due to the association directly with chlorophyll contents^129^*.* Toxic heavy metals, when accumulated above certain permitted levels, adversely influence the bulk and physiological activities of microbiota, soil dynamics, and fertility, finally resulting in a decline in the yield of wheat and, via the food chain, human and animal health. It typically inhibits cation exchange in roots such as K^**+**^, Ca^**2+**^, Mg^**2+**^, Fe^**2+**^, and Zn^**2+**^ and also restricts root absorption sites’ access to vital elements^130^. The depressive effects of cadmium and lead on minerals content may be attributed to the reduction of their uptake. Endophytic fungi may enhance host defense and competitive abilities by increasing efficacious germination and growth rate or enhancing the absorption of nutritional elements by the plant^131^**.** Endophytic fungi boost plant development during metal toxicity by enhancing water and nutrient uptake of the plant, either indirectly by adjusting the host’s metal tolerance or directly by upregulating particular metal tolerance pathways^132^. Also, Foster et al.^133^ declared the ability of EPSs of *Rhizobium etli* to bind to metal ions. Slaveykova and Startchev^134^ verified Cd^**2+**^ complexation by extracellular polymeric substances (EPS) in *S. meliloti*.

Lead accumulation in roots, shoots, and grains of wheat plants increased with increasing external metal concentrations. The present work proved that inoculation with endophytic lead tolerant *A. flavus AUMC* 16,068 and its respective EPS improved Pb accumulation in root, shoot, and grains of wheat plants in comparable to plants grown under Pb stress deprived of inoculation (Fig. 10). The findings of our results were similar to the achievements of^135–137^**.** The roots, which were the first to be exposed to harmful metals, accumulated more metal than the plant shoots^138^. Contrary, under heavy metal stress, rice plants accumulated more Cd and Zn in comparison to maize leaves, which accumulated less Cd and Zn^66^. Lead is likely constrained in roots because it clings to ion-exchangeable sites on the cell wall, resulting in extracellular precipitation as Pb^**2+**^ carbonates^129^. The larger surface area offered by the endophyte, as well as retention of Pb^**2+**^ by the fungal mycelia as a crucial sink for heavy metals via chelation of Pb^**2+**^ ions inside the fungus or adhesion of Pb^**2+**^ to chitin in the wall of the fungal cell, appear to be related to Pb^**2+**^ accumulation in the root and reduced transfer of Pb^**2+**^ from the root to the shoot^139^. Exopolysaccharides offer a defense against several external stressors, including metallic substances^140^. Furthermore, EPS provides nutrients and stores carbon to assist plant growth^109,141^. According to Bhagat et al.^142^, the 3rd environmental stressor is the accumulation of heavy metals in the soil due to inappropriate industrial waste disposal techniques, which are hazardous to plants. Exopolysaccharide synthesis by soil microbes might ultimately result in metal ion biomineralization, providing plants with metal stress resistance. Furthermore, Plant-associated microorganisms create EPS and biosurfactants that aid in their battle against toxic metals ^143^. Also, Mishra et al. ^144^ found that polysaccharide eliminates cadmium, lead, zinc, uranium, copper, and iron aluminum from rhizosphere soil via metal ion adsorption due to the existence of anionic functional groups that involve uronic acids, phosphate, hydroxyl, and succinyl. The susceptibility of EPS to heavy metals is attributed to electrostatic interactions between heavy metal ions and EPS surface functional groups. When it binds to cationic metals such as Cd^**2+**^, Co^**2+**^, Pb^**2+**^, and Ni^**2+**^, it forms EPS-metal complexes^140^**.**

## Conclusion

Agricultural efficiency and harvest rates can be influenced by a variety of environmental stressors such as heavy metals, drought, salt, and high temperatures, all of which have negative impacts on plant growth and development which ultimately results in global food scarcity. Endophytic fungi are a prospective source of natural fertilizers, growth regulators, and natural antimicrobials that can be employed as green alternatives to agrochemicals. The current study identified endophytic *Aspergillus flavus* AUMC 16,068 that provides metal stress tolerance characteristics to plants via EPS production, promotes plant growth, and increases nutrient uptake of wheat plants. This work highlights the potential role of *A. flavus* AUMC 16,068 and its EPS to alleviate the adverse effects of Lead toxicity on the growth of wheat seedlings, through improving growth parameters and antioxidant activities. This study provides a sustainable supply that is easy to cultivate and has a high plant growth-promoting activity. The plant growth-promoting abilities of endophytic fungus, and recent advances in our knowledge of some of the mechanisms, imply that this interesting source warrants additional exploration for possible future use in agriculture.

## Data Availability

The datasets generated and/or analyzed during the current study are available in the Gene Bank database repository, under accession number OQ931875.
